# Anti-tumor activity of metformin: from metabolic and epigenetic perspectives

**DOI:** 10.18632/oncotarget.13639

**Published:** 2016-11-26

**Authors:** Xilan Yu, Wuxiang Mao, Yansheng Zhai, Chong Tong, Min Liu, Lixin Ma, Xiaolan Yu, Shanshan Li

**Affiliations:** ^1^ Hubei Collaborative Innovation Center for Green Transformation of Bio-Resources, College of Life Sciences, Hubei University, Wuhan, Hubei, China

**Keywords:** metformin, therapeutic targets, metabolism, epigenetic modifications

## Abstract

Metformin has been used to treat type 2 diabetes for over 50 years. Epidemiological, preclinical and clinical studies suggest that metformin treatment reduces cancer incidence in diabetes patients. Due to its potential as an anti-cancer agent and its low cost, metformin has gained intense research interest. Its traditional anti-cancer mechanisms involve both indirect and direct insulin-dependent pathways. Here, we discussed the anti-tumor mechanism of metformin from the aspects of cell metabolism and epigenetic modifications. The effects of metformin on anti-cancer immunity and apoptosis were also described. Understanding these mechanisms will shed lights on application of metformin in clinical trials and development of anti-cancer therapy.

## INTRODUCTION

**Figure 1:**
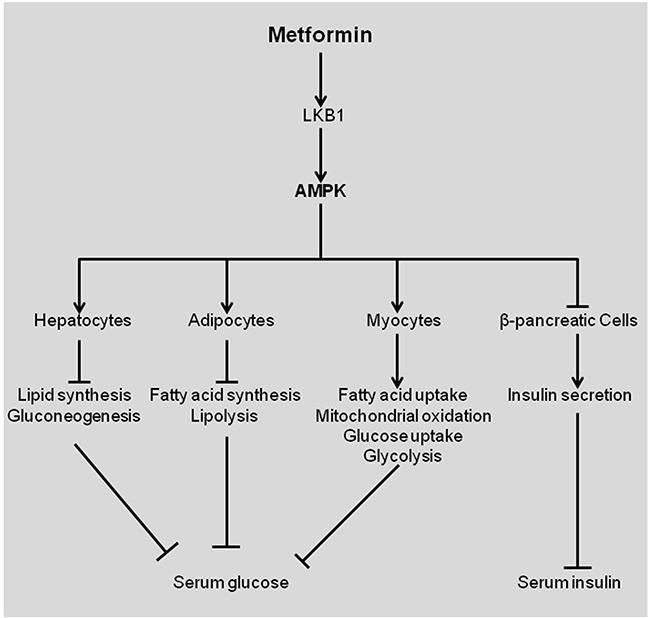
Effects of metformin in patients with diabetes type 2 Metformin activates LKB1, which then activates AMPK, resulting in differential effects in various tissues. Figures were adapted from [2]. AMPK, AMP-activated protein kinase.

Metformin is the world's widely prescribed agent for treatment of type 2 diabetes. Type 2 diabetes is a metabolic disease characterized by reduced tissue responsiveness to insulin, known as insulin resistance [1, 2]. This leads to insufficient peripheral glucose elimination, impaired inhibition of hepatic gluconeogenesis and elevated hepatic glucose output. The anti-diabetic effect of metformin is primarily reflected in hepatocytes, myocytes, adipocytes, and β-pancreatic cells (Figure 1). Metformin inhibits glucose production in liver whereas increases insulin sensitivity in the peripheral tissues, resulting in elevated glucose uptake and consumption by skeletal muscle and adipose tissues (Figure 1) [1]. Metformin treatment reduces insulin secretion by β-pancreatic cells [2]. The key molecule that executes these functions is AMP-activated protein kinase (AMPK), a serine-threonine kinase regulating cellular energy metabolism (Figure 1). Activated AMPK impairs the overall synthesis of proteins, fatty acids, and cholesterol, but increases fatty acids usage [3].

Over the past decade, meta-analysis revealed that metformin-treated diabetic patients tend to have a reduced incidence of cancer [3]. The first evidence came from the report by Evans et al., who found an inverse relationship between metformin usage and cancer incidence for patients with type 2 diabetes [4]. Further studies with type 2 diabetes patients demonstrated that metformin reduced their risk of cancer at many sites, especially for breast, colorectal, ovarian and endometrial cancer [5]. The anti-cancer activity of metformin has also been shown for several cancers in animal models. Kisfalvi et al. reported that administration of metformin significantly reduced the growth of human pancreatic cancer cells xenografted on the flank of nude mice [6]. In addition, metformin significantly inhibits the colony formation and tumor growth in prostate, lung, or colon cancer cell xenografts [7–9]. Metformin has being used in clinical trials in cancer and is now extending to non-diabetic population [5, 10]. In this review, we focused on discussing the potential mechanisms of metformin in cancer prevention and treatment.

## CLASSIC ANTI-CANCER MECHANISMS OF METFORMIN

### Direct or insulin-independent anti-cancer mechanisms of metformin

**Figure 2:**
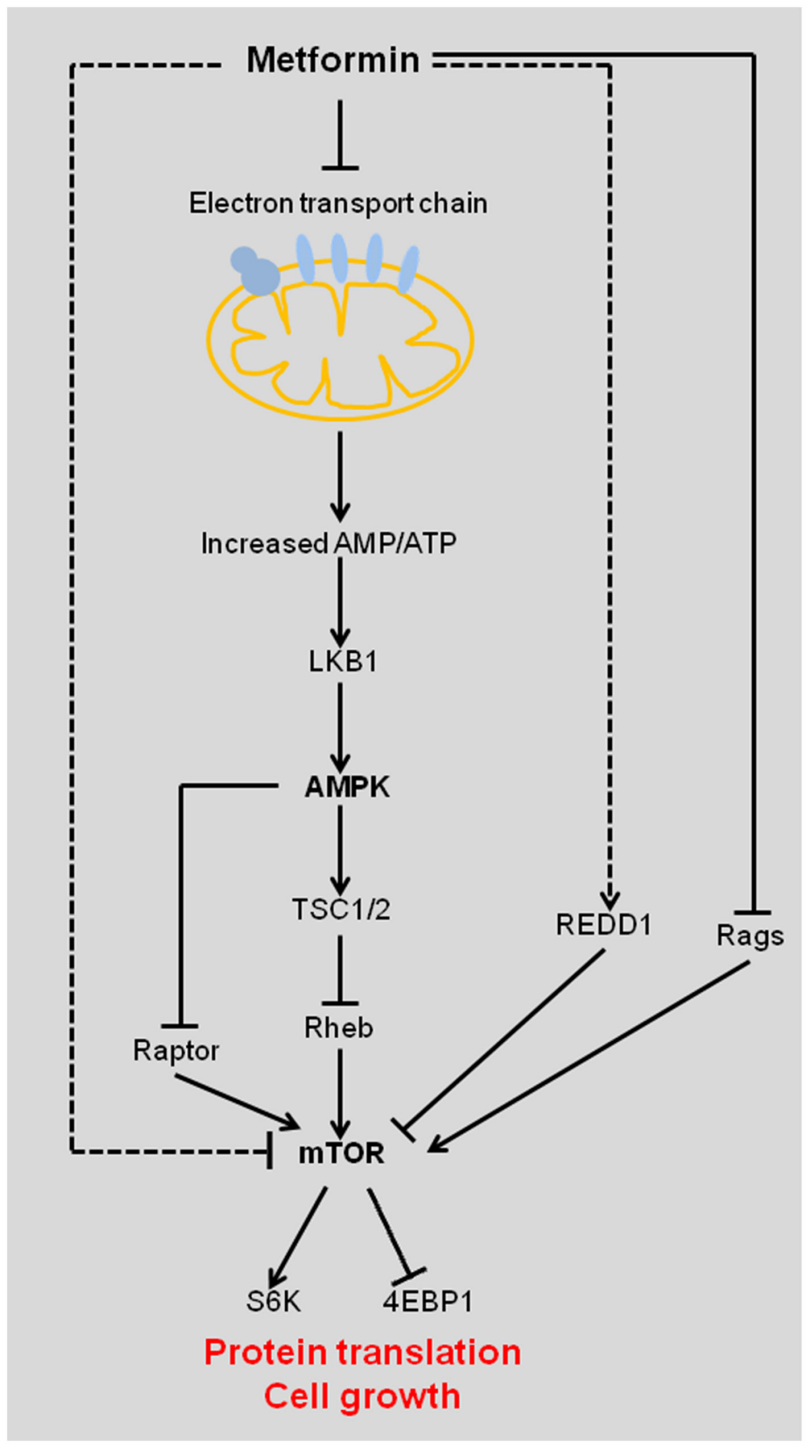
Diagram showing the indirect effect of metformin in suppressing tumorigenesis Metformin inhibits complex I of the electron transport chain, which leads to increased AMP/ATP ratio and activation of AMPK by LKB1. Activated AMPK inhibits mTOR and its downstream targets by the following two pathways: 1. AMPK stabilizes TSC1/2, which inhibits Rheb, an activator of mTOR; 2. AMPK inhibits mTOR binding protein Raptor. Metformin directly inhibits mTOR by up-regulating REDD1 and suppressing Rags. AMPK, AMP-activated protein kinase; Rheb, Ras homolog enriched in brain; LKB1, liver kinase B1; REDD1, regulated in development and DNA damage response 1; TSC, tuberous sclerosis complex; Rags, Rag GTPases; mTOR, mammalian target of rapamycin; 4EBP1, eukaryotic initiation factor 4E binding protein 1; S6K, S6 kinase.

The direct anti-cancer mechanism of metformin is activating the LBK1-AMPK signaling pathway (Figure 2). Metformin uncouples the electron transport chain at complex I, leading to impaired mitochondrial function, decreased adenosine triphosphate (ATP) synthesis, and elevated cellular AMP/ATP ratio [11]. Increased binding of AMP to AMPK activates AMPK by inducing phosphorylation of its catalytic subunit at residue Thr172 by liver kinase B1 (LKB1). LKB1 is a tumor suppressor and its mutations are associated with the Peutz-Jeghers cancer predisposition syndrome [12]. Binding of AMP to AMPK also prevents dephosphorylation of AMPK Thr172 by protein phosphatases. LKB1-activated AMPK phosphorylates and activates the tumor suppressor tuberous sclerosis complex 1 and 2 (TSC1/2), which negatively regulates the activity of mTOR by inhibiting Ras homolog enriched in brain (Rheb) [13]. mTOR is a critical mediator of the phosphatidylinositol-3-kinase/protein kinase B/Akt (PI3K/PKB/Akt) signaling pathway, which is one of the most frequently deregulated molecular networks in human cancer [14, 15]. Metformin-activated AMPK inhibits mTOR and reduces the phosphorylation of its downstream targets, the eukaryotic initiation factor 4E-binding proteins (4EBPs) and ribosomal S6 kinases (S6Ks), leading to an inhibition of global protein synthesis, cell cycle progression, cell proliferation and angiogenesis [2, 5]. Moreover, AMPK has been reported to suppress mTOR signaling pathway independent of TSC2 via phosphorylation of mTOR binding protein Raptor (Figure 2) [16].

Metformin also exerts anti-tumor effects independently of AMPK/LBK1/TSC signaling pathway. Metformin inhibits the mTOR signaling pathway by suppressing Rag GTPase-dependent activation of mTOR (Figure 2) [17]. Moreover, metformin inhibits mTOR independently of AMPK by up-regulating the expression of REDD1 to cause cell cycle arrest [18].

A recent study showed that in pancreatic cancer cells, metformin potentiates the anti-tumor activity of resveratrol by inhibiting vascular endothelial growth factor b (VEGF-B) signaling pathway [19]. Resveratrol has a dual role in pancreatic cancer cells: on one hand, it acts as a tumor suppressor by accelerating apoptosis; on the other hand, it promotes tumorigenesis by up-regulating the expression of VEGF-B, which then activates GSK-3β and inhibits apoptosis [20]. Metformin suppresses resveratrol-induced expression of VEGF-B, thereby potentiating the anti-cancer effect of resveratrol via inhibition of VEGF-B/GSK-3β signaling pathway [19].

### Indirect or insulin-dependent anti-cancer mechanisms of metformin

**Figure 3:**
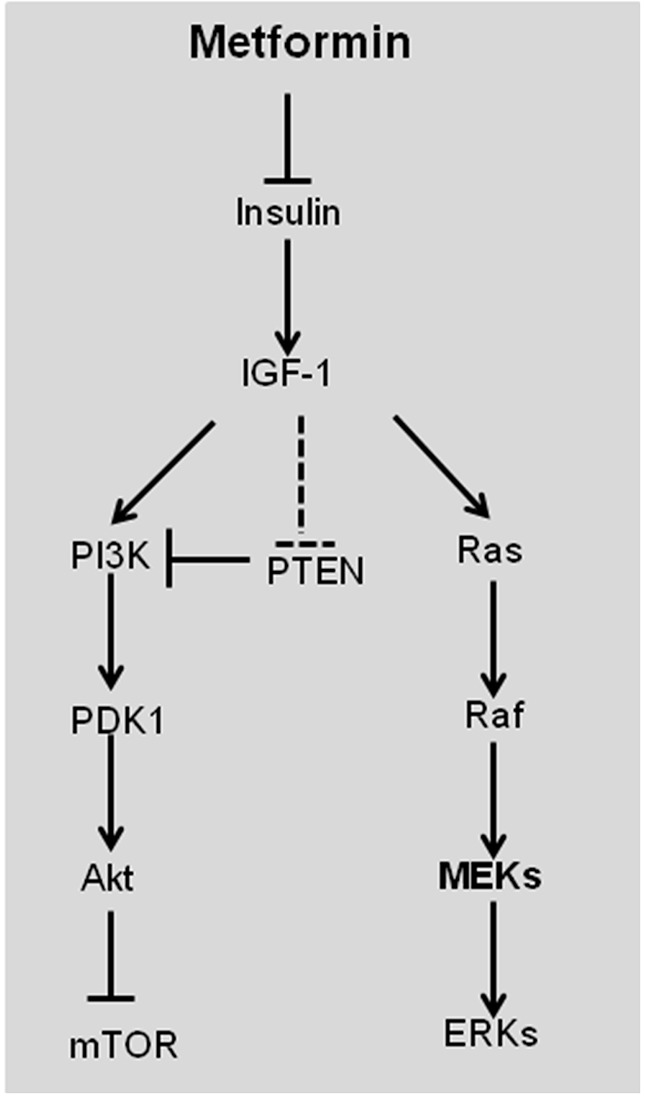
Schematic representation of the direct effect of metformin in suppressing tumorigenesis Insulin increases free IGF-1 levels by displacing IGF-1 from its binding protein. IGF-1 stimulates tumorigenesis by Ras/Raf/MEK/ERK and PI3K/PDK1/Akt/mTOR signal pathways. IGF-1 also negatively regulates PTEN, which inhibits PI3K/PDK1/Akt/mTOR signal pathway. Metformin reduces insulin and IGF-1 to inhibit cell proliferation and survival. IGF-1, insulin-like growth factor-1; PI3K, phosphoinositide 3-kinase; PDK1, phosphoinositide-dependent kinase 1; PTEN, phosphatase and tensin homolog.

The indirect mechanism of metformin in anti-cancer function is related to its ability to lower insulin and insulin-like growth factor 1 (IGF-1) (Figure 3). Insulin increases glucose utilization, which facilitates tumor initiation and progression [5]. Exposure to elevated insulin increased the incidence of liver cancer and heptacellular carcinoma (HCC) [1]. IGF-1 is a more potent mitogen than insulin and, like insulin, binds to its IGF-binding protein (IGFBP) to stimulate cell growth and protect cells from apoptosis via Ras/Raf/MEK/ERK and PI3K/Akt/mTOR signaling pathways (Figure 3) [5]. IGF-1 also inhibits PTEN, which negatively regulates the PI3K/Akt/mTOR signaling pathway. Elevated insulin competes IGF-binding protein (IGFBP) with IGF-1, resulting in dissociation of IGF-1 from IGFBP to stimulate cell growth. Metformin reduces the overall insulin levels, which subsequently lowers free IGF-1 (Figure 3), thereby hindering cell growth [21]. The indirect mechanism of metformin in anti-cancer is primarily prosecuted in liver, where metformin inhibits hepatic gluconeogenesis [1].

## IMMUNE-MEDIATED ANTI-TUMOR EFFECT BY METFORMIN

Metformin can exert anti-tumor activity by enhancing CD8^+^ T cells [22, 23]. CD8^+^ T cells are key players in mediating immunity to tumors [24]. When stimulated with cancer specific antigen, CD8^+^ T cells initiate expansion and differentiation to effector cytotoxic T lymphocytes (CTLs). When antigen is cleared, most effector CD8^+^ T cells die followed by survival of long-lived memory cells [24]. Persistent stimulation by cancer cells makes CD8^+^ T cells lose the ability to secrete cytokines and undergo apoptosis, a process known as immune exhaustion. Pearce et al. reported that metformin increased CD8^+^ long-lived memory cells to protect the mice vaccinated with attenuated *Listeria monocytogenes* expressing OVA from challenge by OVA-expressing tumor cells [23]. Eikawa et al. found that metformin can inhibit apoptosis of CD8^+^ tumor-infiltrating lymphocytes (TILs) and prevent immune exhaustion [22]. Metformin restored its multifunctionality of CD8^+^ TILs by shifting central memory T cells (TCM) to effector memory T cells (TEM), therefore conferring resistant to rechallenge with the same tumor cells [22].

## IMPACT OF METFORMIN ON CELLULAR METABOLISM

### Effect of metformin on glycolysis and tricarboxylic acid (TCA) cycle

Cancer cells have distinct metabolism with normal cells. They have increased glycolysis but reduced oxidative phosphorylation, a phenomena known as “Warburg effect”. The advantage of accelerated glycolysis in cancer cells is accumulation of sufficient amount of macromolecule intermediates that are critical for their survival and proliferation [25]. These macromolecules include ATP, nucleotides, lipids, as well as reduced nicotinamide adenine dinucleotide phosphate (NADPH) for macromolecular synthesis and redox homeostasis [26].

**Figure 4:**
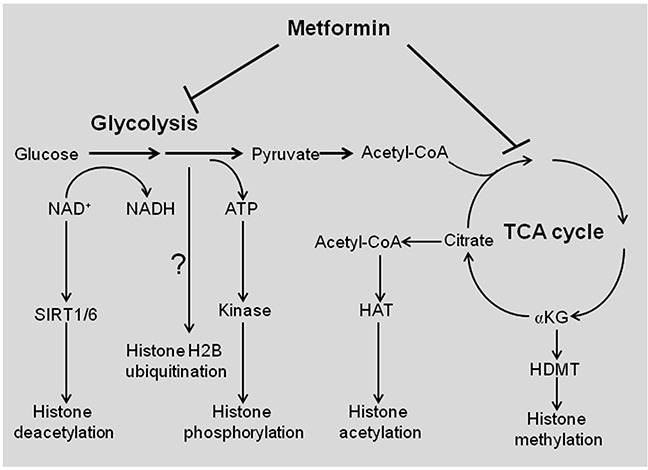
Schematic representation of metabolism-controlled histone modifications by metformin Glycolysis determines the NAD^+^/NADH ratio, which enhances the activity of histone deacetylases (sirtuin) to reduce histone acetylation. Glycolysis provides ATP for protein kinase to phosphorylate histones. The TCA cycle intermediate citrate is converted to acetyl-CoA, which is used for HAT-mediated histone acetylation. Another TCA intermediate αKG is used as cofactor to enhance the ability of HDMT to demethylate histones. Glycolysis is required for H2B ubiquitination, but it is unknown if metformin reduces H2B ubiquitination via blocking glycolysis. HAT, histone acetyltransferase; HDMT, histone demethylase; αKG, α-ketoglutarate.

As a metabolism-control drug, one anti-cancer mechanism of metformin is interfering with cellular metabolism (Figure 4). The prominent change caused by metformin is increased glucose uptake, reduced accumulation of glycolytic intermediates at a specific stage of the pathway, and coordinately decreased TCA cycle intermediates including succinate, fumarate, malate, citrate, and α-ketoglutarate [27, 28]. Nonetheless, it should be noted that in breast cancer stem cells, biguanides only have a modest effect on glycolytic and TCA cycle intermediates, but they strongly deplete nucleotide triphosphates and may hinder nucleotide biosynthesis [28]. Hence, the effect of metformin on cancer metabolism differs depending on the cellular transformation stage [28].

### Effect of metformin on vitamin B12

In addition to glycolysis and TCA cycle, metformin has been shown to reduce the cellular level of folate and vitamin B12 in patients with type 2 diabetes [29, 30]. This effect may enhance the anti-tumor efficacy of metformin, as vitamin B12 deficiency has been reported to inhibit tumor cells in vitro and increase tissue toxicity in patients receiving adjuvant chemotherapy [2, 31]. Nonetheless, this mechanism deserves further attention in the clinical situation, since vitamin B12 deficiency has detrimental effects including neuropathy and anemia [2].

### Is metformin equal to dietary restriction?

Dietary restriction (DR) is the controlled reduction of food intake that can improve late-life health and increase lifespan. Several studies demonstrated that metformin treatment recapitulates the effects of DR, including improvement of late-life health and extension of lifespan in organisms ranging from nematodes to rodents and rhesus monkeys [32]. Extensive work done on animals indicate DR has an important role in suppressing certain cancer types [33]. It is uncertain whether metformin's anti-tumor properties are equal to DR.

## EFFECT OF METFORMIN ON EPIGENETIC MODIFICATIONS

Cell metabolism is tightly linked to epigenetic modifications (Figure 4) [34, 35]. Cells adjust their metabolic states in response to extracellular signaling and/or fuel availability by modulating their epigenetic program and gene transcription. The metabolic sensors that transduce the micro-environment changes to epigenetic modifications, are known as epigenetic “writers” or “erasers” [36].

The typical epigenetic modifications are histone modifications, including histone acetylation, methylation, phosphorylation, ubiquitination, etc. These modifications are catalyzed by specific histone modifying complexes, which require key metabolites as the acetyl, methyl, and phosphate donors (Figure 4). For example, chromatin-modifying enzymes consume key metabolites such as acetyl coenzyme A (acetyl-CoA) for acetylation, S-adenosylmethionine for methylation, and ATP for phosphorylation, etc. These enzymes include DNA methyltransferases (DNMTs), histone acetyltransferases (HATs), histone deacetylases (HDACs), histone methyltransferases (HMTs), and histone demethylases (HDMTs) [36]. Such substrates or cofactors are capable of diffusing through nuclear pores, therefore providing a rapid way to deliver metabolic information to nuclear functions. Thus, the cell's metabolic state could be reflected in the epigenetic modifications. In the following sections, we will discuss the impact of metformin on epigenetic modifications from histone and DNA modifications.

### Metformin and H2BK120 ubiquitination

Du et al. reported that metformin reduced the global leves of histone H2B lysine 120 (H2BK120) monoubiquitination and the transcription of its target genes including p21 and cyclin D1, which are regulators of cell cycle [37]. However, it remains to be determined how metformin reduces H2BK120 ubiquitination and whether metformin down-regulates gene expression via H2BK120 ubiquitination. As metformin inhibits glycolysis [28] and glycolysis is required for H2B ubiquitination [38], it is conceivable that metformin could reduce H2B ubiquitination by inhibiting glucose metabolism pathway. Further efforts are required to investigate this possibility.

### Metformin and histone acetyltransferases/deacetylases

Histone acetylation levels are determined by factors including histone acetyltransferases, histone deacetylases, and intracellular acetyl-CoA [39, 40]. Metformin-activated AMPK has been shown to enhance the activity of histone deacetylase SIRT1 to reduce acetylated p53 and expression of p21 [41]. However, in HepG2 cells, low concentration of metformin treatment suppresses the deacetylase activity of SIRT1 on p53, leading to increased p53 acetylation and cell senescence [10]. For histone acetylation, Khan et al. found that metformin inhibited diabetes-associated HDACs activity, thereby increased the acetylation of histone H3 in liver [42]. However, in breast cancer cells, metformin treatment reduced H3K9 acetylation [43]. It is unknown if metformin inhibits histone H3 acetylation by SIRT1. As metformin blocks glycolysis [28] and acetyl-CoA is the downstream product of glycolysis, it will be interesting to investigate whether metformin reduces H3K9 acetylation via acetyl-CoA.

### Metformin and histone methyltransferase

Transcriptome analysis showed that metformin down-regulated the expression of histone methyltransferase multiple myeloma SET domain (MMSET) in prostate cancer cells [44]. siRNA-mediated knockdown of MMSET attenuates cellular migration and invasion [44]. Hence, MMSET knockdown in combination with metformin treatment further reduced the capacity of prostate cancer cells to migrate and invade [44]. Banerjee et al. reported that metformin treatment increased global H3K4me2 but reduced the global levels of H3K27me2 and H3K9me2 despite it is unknown how metformin alters these histone modifications and how the specificity is determined [43]. As metformin has been shown to decrease TCA cycle intermediate α-ketoglutarate [27, 28], which is a co-activator of histone demethyhalses [45], it is possible that metformin affects histone methylation status via α-ketoglutarate.

### Metformin and DNA methylation

Yan et al. showed that metformin increased DNA methylation at the promoter of H19 [44], whose product is a long noncoding RNA implicated in the pathogenesis of diverse human cancers. H19 reduced the availability of let-7, a potent tumor suppressor microRNA that functions to suppress the expression of oncogenes important for cell growth and motility [44]. Metformin induced DNA methylation at H19 promoter to down-regulate H19 expression and hence inhibit tumor cell migration and invasion [44]. Ishikawa et al. reported that metformin increased DNA methylation in insulin gene promoter, which reduced insulin gene transcription [46]. Much work is required for addressing which DNA methyltransferase(s) are responsible for DNA methylation changes and uncovering the underlying mechanisms.

## EFFECT OF METFORMIN ON APOPTOSIS

Emerging evidence indicates that metformin induces apoptosis in several human cancers. Metformin has been shown to promote apoptosis by inducing the expression and/or activation of caspase 3, the key molecule that executes apoptosis [47–49]. Gao et al. reported that metformin induced apoptosis by stimulating the production of reactive oxygen species (ROS) and reducing the mitochondrial membrane potential [50].

P53 is a tumor suppressor involved in DNA-damage repair and cell cycle control. AMPK can phosphorylate and activate p53, which then induces apoptosis of cells that encounter low nutrient conditions. Metformin could promote apoptosis by activating AMPK/p53 pathway [51]. Sun et al. showed that activated AMPK/p53 induces miR-23a, which triggers apoptosis by inhibiting forkhead box protein A1 (FOXA1) in human hepatocellular carcinoma cells [52]. In addition, metformin triggers apoptosis by inducing metabolism reprogramming. Metformin blocks mitochondrial respiratory activity. As a compensatory response, cells need to enhance the p53-dependent fatty acid β-oxidation and glycolysis [8]. However, this metabolic conversion cannot be executed in p53-deficient cancer cells, leading to their apoptosis [8]. Metformin has also been found to reduce cell proliferation and migration in the oral squamous cell carcinoma (OSCC) cell lines probably by reversing Warburg effect [49]. In OSCC cells, metformin reduced the expression of hypoxia-inducible factor 1α (HIF-1α) and increased pyruvate dehydrogenase (PDH) expression under hypoxic conditions [49]. PDH catalyzes the conversion of pyruvate to acetyl-CoA and hypoxic inhibition of PDH activity allows more pyruvate to be converted to lactate, which is necessary for cancer metabolic reprogramming and growth [53]. The up-regulation of pyruvate dehydrogenase by metformin could promote cell apoptosis by attenuating the Warburg effect.

Other functions of metformin in anti-tumor activity include reducing c-myc protein [54], decreasing cell senescence [55], and inhibiting inflammatory response associated with cellular transformation [56]. Due to space limitations, we will not discuss the details here.

## CONCLUSIONS

Metformin has been extensively studied to suppress tumorigenesis by direct and indirect mechanisms. Recently, researchers began to elucidate the anti-tumor functions of metformin from the aspects of cellular metabolism and epigenetic modifications and much progress has been made. Still, a lot of questions remain to be dissolved. Firstly, there is no comprehensive study to investigate the impact of metformin on epigenetic modifications, such as histone modifications, DNA and/or RNA methylation, noncoding RNA, etc. Secondly, it is unknown whether metformin affects chromatin structure, histone chaperones, etc. Lastly, how does metformin differentially regulate histone modifications, i.e. H3K4me2 versus H3K27me2? What are the key molecules that sense metformin and then transduce the signal to histone modifying enzymes?

The number of clinical trials with metformin in cancer continues to grow into the hundreds [5]. Most of these trials involve metformin in combination with other agents, implying that metformin alone is unlikely to give significant clinical benefits. Given the fact that metformin functions in cellular metabolism and epigenetic modifications, it is conceivable that a combination of metformin with drugs targeting these pathways will become promising anti-cancer therapy in the future. Indeed, Duo et al. showed that metformin can synergistically enhance the anti-tumor activity of histone deacetylase inhibitor trichostatin A against osteosarcoma cells [57]. The combination of metformin and the conventional chemotherapeutic agent, doxorubicin, kills both cancer stem cells and non-stem cancer cells in culture [58]. This combinatorial therapy effectively inhibits tumor growth in nude mice [58]. Clinical trials using a combination of metformin with traditional anti-cancer drugs have been performed in non-diabetic patients, especially in breast cancer patients [59, 60]. It is anticipated that much more efficient combined therapy will be developed with our understanding about the anti-cancer mechanism of metformin.
